# Evaluation of the Effectiveness of Suicide.ca, Quebec’s Digital Suicide Prevention Strategy Platform: Cross-Sectional Descriptive Study

**DOI:** 10.2196/46195

**Published:** 2024-03-06

**Authors:** Louis-Philippe Côté, Julie Lane

**Affiliations:** 1 Centre for Research and Intervention on Suicide, Ethical Issues and End-of-life practices Université du Québec à Montréal Montreal, QC Canada; 2 Centre RBC d’expertise universitaire en santé mentale Université de Sherbrooke Sherbrooke, QC Canada

**Keywords:** suicide prevention, public health, information and communication technology, digital mental health, helpline, digital strategy, communication technology, information technology, suicide, psychoeducation, mobile app, suicide risk, risk factor, users, mental health, text, website, prevention strategy, prevention, Google Analytics, Canada, Quebec, questionnaire, mobile phone

## Abstract

**Background:**

In 2017, the Quebec government assigned the *Association québécoise de prévention du suicide* (AQPS) to develop a digital suicide prevention strategy (DSPS). The AQPS responded by creating a centralized website that provides information on suicide and mental health, identifies at-risk individuals on the internet, and offers direct crisis intervention support via chat and text.

**Objective:**

This study aims to evaluate the effectiveness of suicide.ca, Quebec’s DSPS platform.

**Methods:**

This study used a cross-sectional descriptive design. The study population comprised internet users from Quebec, Canada, who visited the suicide.ca platform between October 2020 and October 2021. Various data sources, such as Google Analytics, Firebase Console, and Customer Relation Management data, were analyzed to document the use of the platform. To understand the profile of suicide.ca users, frequency analyses were conducted using data from the self-assessment module questionnaires, the intervention service’s triage questionnaire, and the counselors’ intervention reports. The effectiveness of the platform’s promotional activities on social media was assessed by examining traffic peaks. Google Analytics was used to evaluate the effectiveness of AQPS’ strategy for identifying at-risk internet users. The impact of the intervention service was evaluated through an analysis of counselors’ intervention reports and postintervention survey results.

**Results:**

The platform received traffic from a diverse range of sources, with promotional efforts on social media directly contributing to the increased traffic. The requirement of a user account posed a barrier to the use of the mobile app, and a triage question that involved personal information led to a substantial number of dropouts during the intervention service triage. AdWords campaigns and fact sheets addressing suicide risk factors played a crucial role in driving traffic to the platform. With regard to the profile of suicide.ca users, the findings revealed that the platform engaged individuals with diverse levels of suicidal risk. Notably, users of the chat service displayed a higher suicide risk than those who used the self-assessment module. Crisis chat counselors reported a positive impact on approximately half of the contacts, and overall, intervention service users expressed satisfaction with the support they received.

**Conclusions:**

A centralized digital platform can be used to implement a DSPS, effectively reaching the general population, individuals with risk factors for suicide, and those facing suicidal issues.

## Introduction

### Overview

Information and communication technologies (ICTs) are increasingly used for suicide prevention. As demonstrated in a scoping study by Rassy et al [1], ICTs are used in a multitude of universal, selective, and indicated suicide prevention strategies.

As defined by the World Health Organization (WHO) [2], universal prevention strategies are designed to reach a whole population. They aim to optimize health for all and minimize the risk of suicide by removing barriers to care, expanding access to support services, and strengthening protective factors. Universal suicide prevention strategies using ICTs typically aim to promote mental health literacy, health behaviors, and help seeking [1]. They often take the form of psychoeducational websites and web-based awareness campaigns. Selective prevention strategies, in contrast, are aimed at specific groups or communities [2]. They can be developed based on sociodemographic or geographic characteristics or according to the prevalence of risk factors [3]. They may target individuals who do not exhibit suicidal behaviors but who are at risk for suicide [2]. Selective suicide prevention strategies using ICTs focus on identifying suicidal internet users, offering mental health self-management programs, and providing web-based training [1]. These strategies are, for the most part, included in broader programs involving direct contact between distressed individuals and health professionals. Finally, indicated prevention strategies focus on individuals who have suicidal ideation or who have previously attempted suicide [2]. Indicated suicide prevention strategies using ICTs include web-based suicide risk assessment and triage systems, web-based psychotherapy, chat- or text-based crisis interventions, and digital tools to support face-to-face interventions [1].

Despite the vast body of scientific literature discussing the application of ICT in suicide prevention, few studies have addressed how multiple ICT-based interventions can be combined and articulated together in the development of a digital suicide prevention platform [3] or, more broadly, as part of a digital suicide prevention strategy (DSPS). In a context where an increasing number of countries are developing digital health strategies [4], guidelines must be developed to guide organizations that wish to develop a DSPS. To deploy the 3 levels of prevention defined by the WHO (universal, selective, and indicated), it is crucial that these guidelines contain information on the best strategies for reaching the general population, identifying people with risk factors for suicide on the internet, and providing direct support to people struggling with suicidal issues.

Rassy et al [1] discussed ICT-based interventions aimed at a broad audience, including educational websites, web-based awareness campaigns, and social media platforms. They spotlight the *It Gets Better* campaign launched in 2010, primarily directed at bullied teenagers, especially those identifying as gay, to prevent suicide. The campaign involved videos created by gay adults that conveyed the message that these teens’ lives would improve over time. These videos were hosted on an educational website and YouTube channel and extensively shared on social media, accumulating >50 million views [5]. Despite the limited evaluation of the effects of educational websites, web-based awareness campaigns, and social media initiatives, studies have demonstrated promising outcomes in terms of reducing suicidal ideation and behavior as well as enhancing knowledge, attitudes, and help-seeking behavior [1]. As highlighted by Rassy et al [1], further research is required to determine the specific characteristics of these interventions that are associated with positive outcomes. In addition, there is a need to develop effective methods for identifying beneficial websites and social media posts and to inform users about or guide them toward internet content that may be of help to them.

Using Google searches holds promise in identifying individuals at risk of suicide on the internet and guiding them toward beneficial web-based content, as individuals with suicidal ideations tend to search for suicide-related information on the internet [6-8]. Indeed, multiple studies have established a correlation between suicide-related Google searches and suicide rates across diverse populations [9-11]. The findings from these studies suggest that suicide-related Google searches can be used to identify individuals at risk of suicide on the internet, recognize intervention opportunities, and facilitate real-time monitoring of suicide risk at a population level. However, it is important to acknowledge that only a small number of real-world interventions based on Google searches have been developed to date.

An example of such an intervention is the study conducted by Sueki et al [12], in which they implemented a web-based gatekeeping intervention in Japan. This intervention involved targeted web-based advertisements directed at individuals searching for suicide-related terms on the web, redirecting them to an email-based consultation. The results of the study indicated a significant reduction in users’ suicidal ideation 4 weeks after being identified on the internet and redirected to the email-based intervention. Furthermore, Onie et al [13] conducted 2 studies to assess engagement levels with 2 advertisement campaigns on Google targeting individuals contemplating suicide. The researchers noted significant engagement with both campaigns, as evidenced by high click-through and conversion rates. Similarly, Google displays crisis helpline numbers at the top of the search results when a user performs a suicide-related search. To our knowledge, no empirical data on the actual impact of Google initiative on helplines use worldwide have been published to date. In addition, data on the characteristics of individuals who were identified by Google and directed to help services are also unavailable.

Regarding ICT-based interventions aimed at providing direct support to individuals facing suicidal issues, crisis lines that offer text- and chat-based services are likely to be the primary source of assistance for those seeking help on the internet. Research on the use and effectiveness of crisis text- and chat-based services is limited. Studies so far have shown that they seem effective in reducing users’ distress [14-18]. However, they reported quite different results regarding the suicidality of users. For example, evaluation of the Netherlands’ 113Online chat service [16] and the US Lifeline Crisis Chat [16] suggested that >80% of their users were experiencing suicidal thoughts. In contrast, evaluation of crisis text lines in the United States and Australia [17,18] indicated that the proportion of users experiencing suicidality was <30%.

In addition, studies suggest that conducting suicide risk assessments during chat- and text-based interactions poses challenges, resulting in fewer assessments completed by counselors [14,19]. These gaps in suicide risk assessments can impact the identification of callers at risk of suicide and potentially bias the reported statistics on suicidal callers by these services. Consequently, providing an accurate description of suicidality among users of these services becomes challenging. Therefore, it is imperative for crisis lines offering text- and chat-based interventions to establish strategies that enable a precise depiction of service users and an assessment of their level of suicidality, rather than solely relying on intervention reports completed by counselors.

In a similar vein, Zabelski et al [20] emphasized the need to investigate the accessibility of chat and text services for high-risk populations in their policy focus review of suicide prevention research on crisis lines. They suggested that if the effectiveness of these services is established, additional studies should be conducted to better understand the demographics and risk profiles of service users as well as to develop effective strategies to promote these services among high-risk populations.

This brief overview of ICT-based interventions highlights the potential of ICTs in the field of suicide prevention. However, it also underscores the existence of significant knowledge gaps that can have important implications for the development of DSPSs. Moreover, essential information required by mental health resources seeking to incorporate ICT-based interventions into their clinical practices is frequently absent from scholarly articles. For example, the technical aspects of developing, maintaining, and operating digital interventions have rarely been discussed [21,22]. Furthermore, there is a dearth of comprehensive research that adequately examines the implementation characteristics of ICT-based interventions in real-world settings, encompassing both the obstacles and factors that aid their implementation [23,24]. Ultimately, to support well-informed decision-making regarding the development of DSPSs, it is vital to establish whether ICT-based suicide prevention strategies are effectively reaching various target audiences or whether they are reaching the same individuals through different means. Therefore, research is necessary to explore digital platforms that incorporate a variety of ICT-based interventions, evaluate the presence of diverse user profiles on these platforms, and analyze the specific interventions they use.

### Background

In 2017, the Quebec government asked the *Association québécoise de prévention du suicide* (AQPS) to set up a DSPS. This DSPS was intended to inform the population about suicide and mental health resources, identify people at risk for suicide on the internet, and offer web-based intervention to people who rarely use traditional help services.

The AQPS then launched a broad consultation process with Quebec suicide prevention stakeholders [7]. The objective of this consultation was to paint a picture of the current practices and needs regarding the use of digital technologies in suicide prevention in Quebec. In general, the consultation participants stressed the importance of developing new digital services by building on the programs, services, and intervention practices already in place in Quebec and of using a centralized website that would make it possible to meet several objectives (inform, identify, and intervene).

In this context, the AQPS, in partnership with its technology partner (the Montreal-based technology firm Nventive), developed the *suicide.ca* digital platform [25]. Suicide.ca is the official digital platform of the Quebec’s DSPS. Through this platform, a multitude of universal, selective, and indicated suicide prevention strategies are deployed.

### Objectives

This study illustrates, through the example of suicide.ca, how a single digital platform can be used to reach the general population, identify people with risk factors for suicide on the internet, and provide direct support to people dealing with suicidal issues. Specifically, this study aims to document the use of the suicide.ca platform; describe the profile of users of the suicide.ca platform; and estimate the effectiveness of universal, selective, and indicated prevention strategies deployed via the suicide.ca platform.

## Methods

### Suicide.ca Platform

This section describes the universal, selective, and indicated suicide prevention strategies implemented through the suicide.ca platform.

#### Suicide.ca’s Universal Prevention Strategies

The components of the suicide.ca platform that enable the deployment of universal prevention strategies are its search engine optimization (SEO), the psychoeducation pages, the blog pages, and the promotion of the platform on social media.

##### SEO Strategy

To fulfill its mission, a digital suicide prevention platform must be known to the public and easily found on the internet. This is why positioning itself favorably in search engine results is at the heart of the suicide.ca platform’s promotional strategy. To do so, an SEO strategy was developed. SEO refers to all the strategies implemented to improve the natural referencing of a website on search engines. For example, all the content on the platform was carefully crafted with the explicit objective of prioritizing search engine ranking.

##### Psychoeducation Pages

When the user arrives at the platform’s home page, they are prompted to choose one of the following sections: *I am thinking about suicide*, *I’m worried about someone with suicidal thoughts*, or *I’m grieving a loss by suicide*. The section for suicidal people is presented as a guide to getting better. The content of the psychoeducation pages can be found on the platform [26].

##### Blog Pages

The suicide.ca platform includes a blog. The blog facilitates the regular generation of fresh content, thereby aiding the enhancement of the platform’s natural search engine rankings. It also provides a great deal of flexibility in terms of the topics covered. Content is sometimes developed based on frequent searches by suicide.ca users on search engines.

##### Promotion of the Platform on Social Media

The AQPS promotes the platform via posts on social media. These posts are generally aimed at promoting the intervention service and communicating information found on the platform. Content from the psychoeducation pages and blog pages is regularly used to create new posts.

#### Suicide.ca’s Selective Prevention Strategies

The components of the suicide.ca platform that enable the deployment of selective prevention strategies are the fact sheets on suicide risk factors, AdWords campaigns, the self-assessment module, the self-management tools, and the mobile app.

##### Fact Sheets on Risk Factors Associated With Suicide

The suicide.ca platform includes a section with numerous fact sheets on the risk factors associated with suicide. These fact sheets address mental health problems (eg, depression, anxiety, and schizophrenia) and difficult life situations (eg, relationship problems, money problems, and isolation). These fact sheets have a dual function: to help users cope with their situation and to identify the internet users who present risk factors for suicide on the web.

##### AdWords Campaigns

AdWords is a paid referencing system developed by Google. This allows organizations to promote their services using paid advertisements. These advertisements appear when internet users perform searches with keywords or expressions that are associated with the services offered. The AQPS frequently uses AdWords campaigns to identify internet users who are conducting research related to suicide, depression, or anxiety.

##### Self-Assessment Module

A self-assessment module consisting of French-validated questionnaires (refer to the details in the *Data Collection Methods and Tools* section) is available to users on the *Taking Stock of Your Mental Health* page.

The self-assessment is performed in 2 stages. During the first stage, users are invited to answer questionnaires allowing them to take stock of their suicidal thoughts, to identify the presence of signs of psychological distress, and to evaluate their positive mental health. If the result of this first evaluation reveals that the user is experiencing suicidal ideation, they are invited to contact the platform’s intervention service via a redirection button. If the user is not referred to the intervention service and the results indicate that they may have a mental health disorder (Kessler Psychological Distress Scale [K10] score [27]), additional questionnaires are offered to identify the presence of symptoms related to various mental health disorders.

##### Self-Management Tools and Mobile App

The platform contains a section called My Tools, which includes a safety plan and various mental health self-management tools. To make the various self-management tools accessible to people who do not have a smartphone, they have been developed in the web-mobile format. Therefore, users can access their information via the web or the platform’s mobile app (eg, like on Facebook or Twitter). Consequently, the user must open an account to access the tools. However, a user account is not required to access the intervention service and the self-assessment module.

#### Suicide.ca’s Indicated Prevention Strategies

The components of the suicide.ca platform that enable the deployment of indicated prevention strategies are the chat and text intervention service, the triage system, the suicide risk assessment tools, and the follow-up emails.

##### Intervention Service by Chat and Text Messaging

Suicide.ca is a digital intervention service that operates 24 hours a day, 7 days a week. It is operated by the AQPS and 3 suicide prevention centers. The service can be accessed via the web, the mobile app, or by SMS text messaging.

##### Triage

The intervention service triage system assigns a priority rating to contact requests. The first questions ask about age, gender, and reason for contact. Individuals at the risk of suicide are then asked, *Are you planning to attempt suicide in the coming minutes or hours?* If the user answers *yes*, the triage ends, and they are sent to the queue with a priority rating. If the user answers *no* or *unsure*, they are prompted to complete the Suicidal Ideation Attributes Scale—French version (SIDAS-FR [28]). Finally, the user may, if they wish, specify in a few words the reason for contacting the service.

##### Follow-Up Emails

At the end of the exchange, the counselors can ask users if they would like to receive a follow-up email. These follow-up emails are generally used when the user anticipates a difficult event that could lead to a suicidal crisis (eg, court appearance and meeting with an ex-spouse).

#### Suicide.ca Prevention Model

Using the classification proposed by Rassy et al [1], Textbox 1 illustrates how the different components of the platform are complementary and how they deploy universal, selective, and indicated prevention strategies.

**Suicide.ca prevention model.**

**Universal prevention strategies**

- Search engine optimization
- Psychoeducation pages
- Active promotion of the platform on social media
- Blog pages

**Selective prevention strategies**

- Fact sheets on suicide risk factors
- AdWords campaigns
- Self-assessment
- Self-management tools and mobile app

**Indicated prevention strategies**

- Intervention service by chat and text
- Triage
- Follow-up email

### Study Design

The evaluation of the suicide.ca platform used a cross-sectional design to document the use of the suicide.ca platform; to describe the profile of users; and to estimate the effectiveness of universal, selective, and indicated prevention strategies deployed through the platform.

### Population

The population under study in the evaluation of the suicide.ca platform were the internet users who visited the suicide.ca platform.

### Recruitment

This study was based on the analysis of secondary data collected by AQPS. Data were collected from internet users from Quebec, Canada, who visited the suicide.ca platform between October 2020 and October 2021.

### Data Collection Methods and Tools

#### Overview

The evaluation of the suicide.ca platform was carefully considered during its development. The questionnaires integrated into the platform’s various components made it possible to measure the platform’s impact. The platform collects various types of data.

#### Google Analytics

Google Analytics is a statistical tool offered by Google. It allows website administrators to analyze the behavior of their users. The analytics used in this study are the number of times different pages of the platform were visited, the number of visitors (notwithstanding the number of visits), the sources of traffic acquisition on the platform (eg, search engines, social networks, and hyperlinks on other websites), and the landing pages (the first pages visited by users).

#### Firebase Console

The *Firebase Console* platform is a mobile app design tool supported by Google. It was used to count the number of times the *My Tools* app was downloaded and the number of user accounts created.

#### Self-Assessment Module Questionnaires

The self-assessment module consists of a series of validated questionnaires in French (Table 1). The results of the self-assessments are recorded in the platform’s Customer Relationship Management (CRM).

**Table 1:** Self-assessment questionnaires.

Constructs	Screening questionnaires
Positive mental health	MHC-SF^a^ [29]
Psychological distress	K10^b^ [27]
Suicidal ideation	SIDAS-FR^c^ [28]
Depression	PHQ-9^d^ [30]
Bipolarity	MDQ^e^ [31,32]
Social phobia	SPIN^f^ [33]
Generalized anxiety	PSWQ^g^ [34,35]
Addiction: alcohol	DÉBAA^h^ [36]
Addiction: drugs	DÉBAD^i^ [36]
Addiction: gambling	DÉBA^j^-Jeu [36]
Dependency: screens	DÉBA-internet^k^ [37]
Posttraumatic stress	PCL-5^l^ [38]
Social support	SPS^m^ [39,40]
Reasons to live	RFL^n^ [41,42]

^a^MHC-SF: Mental Health Continuum Short Form.

^b^K10: Kessler Psychological Distress Scale.

^c^SIDAS-FR: Suicidal Ideation Attributes Scale—French version.

^d^PHQ-9: Patient Health Questionnaire-9.

^e^MDQ: Mood Disorder Questionnaire.

^f^SPIN: Spin Phobia Inventory.

^g^PSWQ: Penn State Worry Questionnaire.

^h^DÉBAA: Dépistage et Évaluation du Besoin d’Aide—Alcool.

^i^DÉBAD: Dépistage et Évaluation du Besoin d’Aide—Drogues.

^j^DÉBA-Jeu: Dépistage et Évaluation du Besoin d’Aide—Jeu.

^k^DEBA-internet: Dépistage et Évaluation du Besoin d’Aide—internet.

^l^PCL-5: PTSD Checklist for DSM-5.

^m^SPS: Social Provisions Scale.

^n^RFL: Reasons for Living Inventory.

#### Triage Questionnaire

The triage questionnaire includes inquiries about age, gender, reason for contact, whether the individual is planning to attempt suicide in the near future, and the SIDAS-FR. The results of the triage questionnaire are recorded in the platform’s CRM.

#### Intervention Reports

In this study, the data used from the intervention reports were the reasons for contact, the needs and issues discussed with the users, and the users’ suicide risk ratings assigned by the counselors (suicide risk ratings: *no foreseeable risk for a suicide attempt in the near future, low foreseeable risk for a suicide attempt in the near future, high foreseeable risk for a suicide attempt in the near future, and imminent danger of suicide attempt or attempt in progress*). The categories of needs and issues discussed are not mutually exclusive. The intervention report also contains a section where the counselor is asked to indicate whether they think the user feels better and is more able to cope with their difficulties as a result of the intervention (response choice=*yes*, *unsure*, and *no*). These intervention reports are recorded in the platform’s CRM.

#### Postintervention Questionnaire

This questionnaire includes 9 items in the form of 4-point Likert scales (1=*strongly agree* to 4=*strongly disagree*). The items focus on users’ perceptions of the quality of interventions and their impact on distress, feeling of being able to cope with the situation, and suicidal ideation. The items are illustrated in detail in the *Results* section.

### Analysis Methods

For each objective, several data sources were consulted, and different types of analyses were performed.

#### Objective 1: To Document the Use of the Suicide.ca Platform

To document platform use, Google Analytics data from the suicide.ca website [26] were analyzed to describe platform traffic and the number of visits to the psychoeducation pages. The Firebase Console data from the *My Tools* mobile app were analyzed to document the number of downloads of the mobile app and the number of user accounts created. CRM data were analyzed to describe the number of people who completed the self-assessment, the number of people who accessed the triage of the intervention service, and the number of interventions provided.

#### Objective 2: To Describe the Profile of Users of the Suicide.ca Platform

To describe the profile of platform users, frequency analyses were conducted using data from the self-assessment questionnaires, the triage questionnaire, and the reasons for contact recorded in the intervention reports.

#### Objective 3: To Describe the Impact of Universal, Selective, and Indicated Prevention Strategies Deployed Through the Suicide.ca Platform

The impact of the platform’s promotional activities on social media was investigated through an analysis of the platform’s traffic peaks. First, a visual inspection of the traffic curve was performed using Google Analytics. It was then decided to define the traffic peaks as days with >600 visits. This threshold corresponded to twice the average traffic and days when the increase in traffic was evident in the Google Analytics graph. AQPS internal documentation was then inspected to determine if these dates corresponded to dates when AQPS conducted promotional activities on social media or traditional media (eg, web-based publications, radio, or television interviews). The data analyzed to assess the impact of AQPS promotional activities cover the period from October 15, 2020, to October 17, 2021.

With respect to the AdWords campaigns and the suicide risk factor fact sheets, 2 analytical strategies were used to estimate their effectiveness. The first strategy was to use Google Analytics to describe the traffic acquisition sources of suicide.ca. In digital marketing, traffic acquisition sources identify how visitors to a website accessed a site (eg, via search engine queries, social media posts, AdWord advertisements [43]). Thus, traffic acquisition sources were used to describe the number and proportion of users who arrived at the site through an AdWords campaign. The second strategy was to use Google Analytics to identify the top landing pages on the platform. A landing page is the first page a user views when they arrive on the platform (eg, risk factor information page, psychoeducation page, and resource information page). In digital marketing, landing pages are used to better understand what users were looking for on the internet before they arrived at a site or what caught their attention [43]. For example, landing pages were used to describe the number and proportion of users who began their site visit by viewing a suicide risk factor fact sheet. The conversion rate for the AdWords campaigns was calculated based on the number of individuals who clicked on the advertisement and initiated the crisis chat triage. The analytical data on AdWords campaigns and fact sheets cover the period from December 31, 2020, to October 31, 2021.

The impact of the intervention service was assessed via an analysis of counselors’ Perceptions of the impact of their interventions (recorded in intervention reports) and the postintervention survey results. Approximately 3473 interventions were provided between the postintervention survey going live in late January 2021 and October 2021. Therefore, we estimated the response rate to the postintervention survey to be approximately 10%. As the SMS text messaging intervention service was in development at the time of data collection, only the data for the chat-based intervention are presented.

### Ethical Considerations

This study is based on the secondary analysis of anonymous data collected by AQPS. Consent for primary data collection was obtained through a waiver form in suicide.ca’s self-assessment module and a consent form in the postintervention questionnaire of the intervention service. These forms specify that data collection is for the purpose of continuous service improvement and that any data shared with third parties will be anonymized and subjected to ethics approval by an institutional research ethics committee. The participants were not offered any compensation for their involvement in the study. Ethics approval for the secondary analysis of all data presented in this study was obtained from the institutional review board of the *Centre intégré universitaire de santé et de services sociaux de l’Estrie—Centre hospitalier universitaire de Sherbrooke* (project 2021-4016).

## Results

In this section, the results concerning platform use; the profile of platform users; and the effectiveness of universal, selective, and indicated prevention strategies deployed through the suicide.ca platform are presented.

### Use of the Suicide.ca Platform

#### Overview

A total of 127,495 visits were made to the suicide.ca platform from October 15, 2020, to October 17, 2021. The average number of visits per day was 346.45 (median 313.5, IQR 257-389). The least busy day had 123 visits, and the busiest day had 1774 visits.

#### Visits to Psychoeducation Pages

The number of visits to the psychoeducation pages of the 3 main sections of suicide.ca were much lower than the total number of visits to the site. Moreover, the number of page views dropped significantly after the title pages of all 3 sections were visited (Table 2).

**Table 2:** Suicide.ca’ psychoeducation pages.

Psychoeducation pages			Visits to the page or website (n=127,495), n (%)		Users who visited the page or website (n=100,114), n (%)		Average visit duration (s)
“**I’m thinking about suicide” (title page)**			14,206 (11.55)		10,063 (10.05)		45.37
	Taking stock of your mental health	5312 (4.17)		4478 (4.47)		124.66	
	Taking care of yourself	3148 (2.47)		2642 (2.64)		106.90	
	Finding support services	2051 (1.16)		1778 (1.78)		153.31	
	Talking about it with your loved ones	1238 (0.97)		1098 (1.09)		120.84	
“**I’m worried about someone with suicidal thoughts” (title page)**			9155 (7.18)		7331 (7.32)		54.52
	Recognizing signs of distress	5546 (4.35)		4306 (4.30)		105.44	
	Supporting someone in difficulty	1788 (1.40)		1573 (1.58)		161.44	
	Talking about suicide with someone	1544 (1.21)		1344 (1.34)		161.06	
	Respecting your limits and taking care of yourself	1056 (0.83)		955 (0.95)		124.29	
	Helping someone recover	1012 (0.79)		872 (0.87)		150.34	
“**I’m grieving a loss by suicide” (title page)**			3414 (2.67)		2695 (2.69)		64.80
	Learning about a death by suicide	857 (0.67)		695 (0.69)		77.92	
	Understanding suicide grief	812 (0.63)		653 (0.65)		90.88	
	Suicide grief and children	591 (0.46)		441 (0.44)		28.77	
	When to seek professional help	538 (0.42)		423 (0.42)		113.32	
	Resuming your life after grieving a death by suicide	451 (0.35)		377 (0.37)		129.43	
	Talking about it with family and friends	269 (0.21)		250 (0.25)		154.20	

#### Number of People Who Completed the Self-Assessment Module

With regard to self-assessment, 2488 users completed the SIDAS-FR. Moreover, 3100 users completed the K10 questionnaire, and 2644 users completed the Mental Health Continuum Short Form questionnaire. Overall, 1471 users completed phase 2 of the self-assessment module.

#### Number of Downloads of the Mobile App Versus Number of User Accounts Created

The analysis of downloads of the mobile app and the creation of user accounts was carried out for the period from November 23, 2020, to October 17, 2021. During this period, there were 5159 downloads of the mobile app, and 2178 user accounts were created.

#### Number of Crisis Interventions Provided and Triage Dropout Rate

From October 15, 2020, to October 17, 2021, suicide.ca’s intervention service received 6152 intervention requests. Of these 6152 requests, 1540 (25.02%) did not result in an intervention. The intervention reports provided reasons for not being able to intervene in 1321 of these cases. The most common explanation was the lack of response from the users at the beginning of the contact (840/1321, 63.59%).

**Figure 1:**
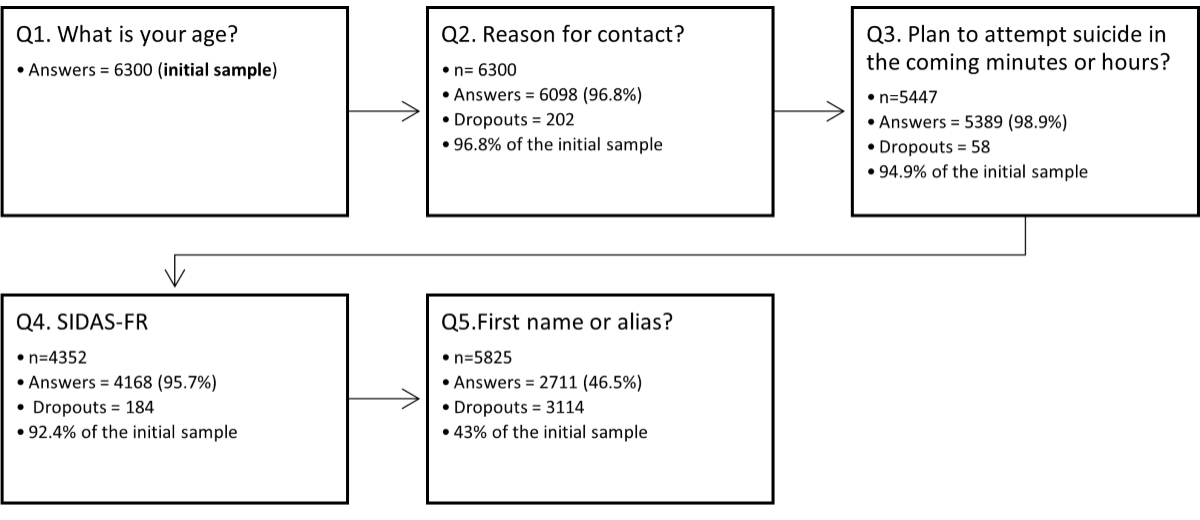
Dropouts at triage (suicidal individuals aged ≥14 y). SIDAS-FR: Suicidal Ideation Attributes Scale—French version.

Analysis of the intervention service triage data was conducted for the period October 15, 2020, through July 25, 2021 (Figure 1). As the gender question was optional, it was not included in the analysis. Of the 6300 users who responded to the first question, only 2711 (42.86%) completed the triage. The last question, which asked for the user’s first name or alias, resulted in a significant dropout rate. Specifically, only 46.54% (2711/5825) of the users who were presented with the question actually provided a response. As for the questions about suicidality, they resulted in few dropouts. Age, reason for contact, and plan to attempt suicide in the coming minutes or hours were not associated with the probability of abandonment during triage.

### Profile of Users of the Suicide.ca Platform

To describe the profile of platform users, frequency analyses were conducted using data from the self-assessment questionnaires, the triage questionnaire, and the reasons for contact recorded in the intervention reports.

#### Characteristics of Individuals Using the Self-Assessment Module

Table 3 indicates the results of the users who undertaken a self-assessment. With regard to suicidality, the average score of the 2488 users who completed the SIDAS-FR questionnaire was 13.47 (SD 14.04; median 10). Among them, 868 (34.89%) reported no suicidal ideation in the last 30 days, whereas 825 (33.16%) reported severe suicidal ideation. In terms of psychological distress, of the 3100 users who completed the K10 questionnaire, 2721 (87.78%) obtained a score indicating the presence of a mental health disorder. With regard to positive mental health, of the 2644 users who completed the Mental Health Continuum Short Form questionnaire, 1024 (38.73%) exhibited languishing mental health.

In the 1471 users who completed phase 2 of the self-assessment, depressive and anxiety disorders were the most prevalent. Specifically, 62.2% (915/1471) of the users obtained a Patient Health Questionnaire-9 score indicating the presence of depression, 61.73% (908/1471) of the users obtained a Penn State Worry Questionnaire score indicating the presence of generalized anxiety disorder, and 53.2% (451/847) of the users obtained a Spin Phobia Inventory score indicating the presence of social phobia disorder.

**Table 3:** Results of the completed self-assessments.

Characteristic			Self-assessment, n (%)
**Severity of suicidal ideation (SIDAS-FR^a^; n=2488)**			
	Absence of suicidal ideation (0)	868 (34.89)	
	Low severity (1-12)	482 (19.37)	
	Medium severity (13-19)	313 (12.58)	
	High severity (>20)	825 (33.16)	
**Psychological distress (K10^b^; n=3100)**			
	Probable presence of a mental health problem	2721 (87.77)	
**Positive mental health (MHC-SF^c^; n=2644)**			
	Flourishing mental health	256 (9.68)	
	Moderate mental health	1364 (51.59)	
	Languishing mental health	1024 (38.73)	
**Psychological disorders (n=1471)**			
	Depression (PHQ-9^d^)	915 (62.2)	
	Bipolarity (MDQ^e^)	89 (6.05)	
	Social phobia (SPIN^f^; n=847)	451 (53.2)	
	Generalized anxiety (PSWQ^g^)	908 (61.73)	
	Addiction: alcohol (DÉBBAA^h^)	149 (10.13)	
	Addiction: drugs (DÉBAD^i^)	246 (16.72)	
	Addiction: gambling (DÉBA^j^-Jeu)	30 (2.04)	
	Addiction: screens (DÉBA-internet^k^)	320 (21.75)	
	Posttraumatic disorder (PCL-5^l^)	414 (28.14)	

^a^SIDAS-FR: Suicidal Ideation Attributes Scale—French version.

^b^K10: Kessler Psychological Distress Scale.

^c^MHC-SF: Mental Health Continuum Short Form.

^d^PHQ-9: Patient Health Questionnaire.

^e^MDQ: Mood Disorder Questionnaire.

^f^SPIN: Spin Phobia Inventory.

^g^PSWQ: Penn State Worry Questionnaire.

^h^DÉBBAA: Dépistage et Évaluation du Besoin d’Aide—Alcool.

^i^DÉBAD: Dépistage et Évaluation du Besoin d’Aide—Drogues.

^j^DÉBA: Dépistage et Évaluation du Besoin d’Aide—Jeu.

^k^DÉBA-internet: Dépistage et Évaluation du Besoin d’Aide—internet.

^l^PCL-5: PTSD Checklist for DSM-5.

#### Characteristics of Individuals Using the Intervention Service

Table 4 presents information on the age and gender of intervention service users. Information on user age was available for 4000 interventions. Most users were aged between 18 and 40 years (2829/4000, 70.73%). Information on the gender identity of users was available for 3569 interventions. Most users identified as woman (2555/3569, 71.59%). Users who identified as transgender, nonbinary, or listed *other* were generally young people aged ≤25 years (235/280, 83.9%).

Information on the reason for contact was available for 4000 interventions (Table 5): 84.48% (3379/4000) of the users indicated *I am thinking about suicide* in the triage questionnaire and 10.1% (404/4000) of the users indicated *I am worried about a loved one*, and 5.43% (217/4000) indicated *I am bereaved by suicide*.

**Table 4:** Sociodemographic profile of the intervention service users.

Age group (y)	Gender identity, n (%)		
	Woman (n=2555)	Man (n=734)	Transgender, nonbinary, or other (n=280)
≤17	211 (8.26)	106 (14.4)	76 (27.1)
18-25	1002 (39.22)	232 (31.6)	159 (56.8)
26-40	1017 (39.8)	213 (29)	43 (15.4)
≥41	325 (12.72)	183 (24.9)	2 (0.7)

**Table 5:** Reason for contact of the intervention service users.

Characteristic		Reason for contact		
		Thinking about suicide	Worried about someone	Grieving a loss by suicide
**Age groups (y), n (%)**				
	≤17	580 (17.16)	52 (12.9)	21 (9.7)
	18-25	1251 (37.02)	119 (29.5)	95 (43.8)
	26-40	1196 (35.39)	127 (31.4)	39 (18)
	≥41	352 (10.42)	106 (26.2)	62 (28.6)
	Total	3379 (100)	404 (100)	217 (100)
**Gender identity, n (%)**				
	Woman	2178 (73.06)	250 (64.8)	127 (62.9)
	Man	555 (18.62)	119 (30.8)	60 (29.7)
	Transgender, nonbinary, or other	248 (8.32)	17 (4.4)	15 (7.4)
	Total	2981 (100)	386 (100)	202 (100)

With regard to the suicidal risk of the users of the intervention service, 3490 answered the question (Table 6), *Are you planning to attempt suicide in the coming minutes or hours?* Of the 3490 users, 1800 (51.58%) answered *no*, 972 (27.79%) answered *unsure*, and 718 (20.57%) answered *yes*. A total of 2606 users completed the SIDAS-FR at triage. The mean score was 26.69 (SD 12.79; median 29). Overall, 75.56% (1969/2606) of the users had suicidal ideation of high severity.

Information on the needs expressed by the users was available for 3254 interventions (Table 6). Overall, 75.29% (2450/3254) of the users expressed a need to ventilate. The most frequently discussed issues were suicide (3033/3475, 87.28%), mental health problems (765/3475, 22.01%), emotional problems (657/3475, 18.91%), family problems (455/3475, 13.09%), self-harm (413/3475, 11.88%), loneliness (407/3475, 11.71%), and problems with peers or relatives (361/3475, 10.39%).

Information on the counselors’ suicide risk assessments was available for 1783 interventions (Table 6). The most common assessments were “Low foreseeable risk for a suicide attempt in the near future” (955/1783, 53.56%) and “High foreseeable risk for a suicide attempt in the near future” (602/1783, 33.76%).

**Table 6:** Intervention service users’ suicide risk and presenting problems.

Characteristic			Suicidal users, n (%)
**Plan to attempt suicide in the coming minutes or hours? (n=3490)**			
	No	1800 (51.57)	
	Unsure	972 (27.85)	
	Yes	718 (20.57)	
**Severity of suicidal ideation (SIDAS-FR^a^; n=2606)**			
	Absence of suicidal ideation (0)	267 (10.24)	
	Low severity (1-12)	120 (4.6)	
	Medium severity (13-19)	250 (9.59)	
	High severity (>20)	1969 (75.56)	
**Expressed needs (n=3254)**			
	Need to ventilate	2450 (75.29)	
	Support for a crisis situation	1093 (33.59)	
	Help to ensure safety	870 (26.74)	
	Ask for advice	741 (22.77)	
	Ask for information	317 (9.74)	
**Issues discussed (n=3475)**			
	Suicide	3033 (87.28)	
	Mental health	767 (22.07)	
	Emotional problems	657 (18.91)	
	Family problems	455 (13.09)	
	Self-harm	413 (11.88)	
	Loneliness	407 (11.71)	
	Problems with peers or relatives	361 (10.39)	
	School problems	294 (8.46)	
	Death of a loved one	293 (8.43)	
	Physical health	179 (5.15)	
	Alcohol or substance abuse	165 (4.75)	
	Verbal abuse survivor	95 (2.73)	
	Perpetrator of physical or verbal abuse	70 (2.01)	
	Bullying at school	68 (1.96)	
	Problems with the law	44 (1.27)	
	Bullying in the workplace	39 (1.12)	
	Pregnancy or abortion	34 (0.98)	
	Other types of problems	357 (10.27)	
**Counselors’ suicide risk assessment (n=1783)**			
	No foreseeable risk for a suicide attempt in the near future	123 (6.89)	
	Low foreseeable risk for a suicide attempt in the near future	955 (53.56)	
	High foreseeable risk for a suicide attempt in the near future	602 (33.76)	
	Imminent danger of suicide attempt or attempt in progress	103 (5.77)	

^a^SIDAS-FR: Suicidal Ideation Attributes Scale—French version.

### Effectiveness of Universal, Selective, and Indicated Prevention Strategies Deployed Through the Suicide.ca Platform

#### Impact of Promotional Activities on the Platform’s Traffic

**Figure 2:**
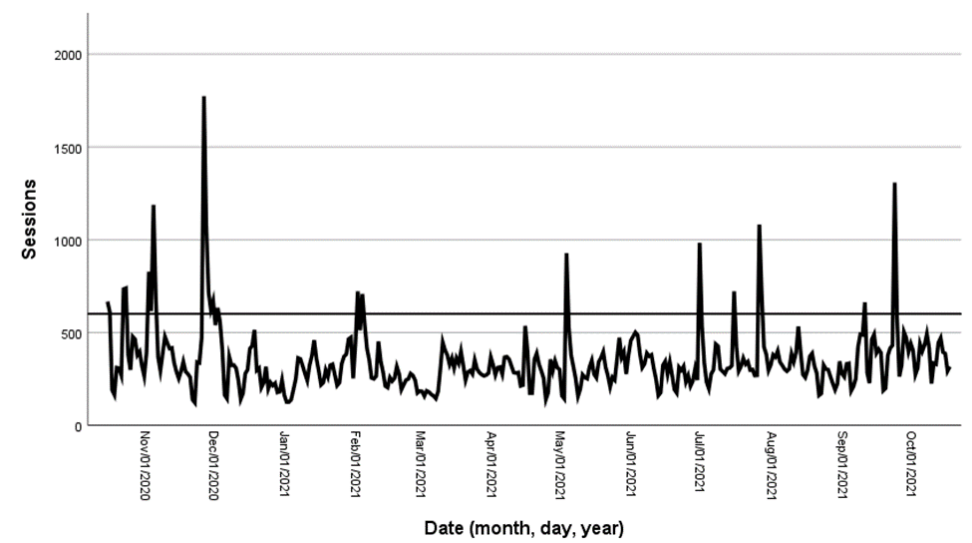
Impact of the promotional activities on the platform’s traffic.

Analysis of peak traffic (days with >600 visits) for the period from October 15, 2020, to October 17, 2021, indicated that they were primarily associated with promotion of the platform in traditional media and on social media (Figure 2; Table 7). Traffic tended to remain high for 1 or 2 days following these spikes and then returned to its average trend afterward.

**Table 7:** Promotional activities associated with traffic peaks.

Date	Visits to the website, n	Activities or events
October 15, 2020	667	Publication on social media
October 22, 2020	734	Publication on social media
November 2, 2020	828	Unknown event (traffic mostly acquired via social media)
November 4, 2020	1188	Unknown event (traffic mostly acquired via direct acquisition)
November 26, 2020	1774	Several publications and interviews about suicide.ca on traditional media
February 1, 2021	722	Suicide Prevention Week
February 3, 2021	708	Suicide Prevention Week
May 3, 2021	928	Publication on social media
June 30, 2021	984	Publication on social media
July 15, 2021	722	Mass emailing to organizations to promote suicide.ca
July 26, 2021	1082	Publication on social media
September 10, 2021	663	World Suicide Prevention Day
September 23, 2021	1308	Publication on social media

#### Impact of AdWords Campaigns and Suicide Risk Factors Fact Sheets on the Platform’s Traffic

Table 8 shows the source of traffic and the top landing pages on the platform for the period from December 31, 2020, to October 31, 2021. The source of traffic to the platform was very diverse. AdWords campaigns on suicide risk factors were the second-largest source of traffic acquisition on the platform.

**Table 8:** Sources of traffic acquisition and landing pages for suicide.ca.

Variable		Website visits attributed to traffic acquisition and landing pages, n (%)
**Main sources of traffic acquisition** **(n=103,402)**		
	Suicide.ca entered directly in the search bar	33,635 (32.53)
	Targeted advertising on search engines (AdWords)^a^	25,251 (24.42)
	Social media	16,775 (16.22)
	Search engines (excluding targeted advertisements)	11,760 (11.37)
	Hyperlink on another website	9899 (9.57)
**Landing pages** **(n=125,677)**		
	Home page	50,271 (40)
	Suicide risk factor fact sheets^a^	20,497 (16.30)
	My Tools (web and mobile app)	13,717 (11)
	Blog pages	12,124 (10.91)
	Pages for contacting the intervention service	9892 (7.87)
	Other	9922 (7.87)
	Section for people who are worried about a loved one	3860 (3.07)
	Section for suicidal people	3056 (2.43)
	Section for people bereaved by suicide	670 (0.53)
	Directory of resources	656 (0.52)

^a^Pages designed to identify internet users at risk for suicide.

The AdWords campaign targeting anxiety and depression was displayed 72,713 times and received 9576 clicks, resulting in a click rate of 13.7%. This campaign also led to 36 crisis chat interventions, representing a conversion rate of 0.38%. Similarly, the AdWords campaign with suicide-specific keywords was displayed 27,961 times and received 2647 clicks, resulting in a click rate of 9.47%. This campaign resulted in 55 crisis chat interventions, with a conversion rate of 1.93%.

As for the first pages consulted by users (Table 8), 40% (4704/11,760) of the visits from search engine queries, excluding AdWords ads, began with a visit to a suicide risk factor fact sheet. Therefore, suicide risk factor fact sheets were an important source of traffic to the platform.

#### Effectiveness of the Intervention Service

Table 9 presents the counselors’ perception of the impact of their interventions. Counselors indicated that the users felt better after the intervention in 56.09% (2565/4573) of the interventions. They also reported that the users felt more able to overcome their difficulties in 51.08% (2336/4573) of the interventions.

**Table 9:** Counselors’ perception of the impact of their interventions (n=4573).

Response	The user feels better, n (%)	The user feels more able to overcome their difficulties, n (%)
No	449 (9.82)	446 (9.75)
Not sure	1559 (34.09)	1771 (38.73)
Yes	2565 (56.09)	2336 (51.08)

Table 10 shows the results of the postintervention survey for 259 suicidal users and 91 users worried for a loved one or bereaved by suicide. Overall, 91.5% (237/259) of the suicidal users indicated that the counselor seemed warm and caring, and 89.5% (232/259) of the users indicated that they felt listened to, understood, and respected. In terms of the effectiveness of the interventions, 82.6% (214/259) of the suicidal users indicated that they felt better, calmer, or more relaxed following the intervention; 76.8% (199/259) of the users indicated that they felt more in control of their suicidal thoughts; and 72.5% (188/259) of the users indicated that they felt more able to overcome their difficulties.

**Table 10:** Respondents who answered somewhat agree or strongly agree to the satisfaction survey.

Satisfaction survey items	Suicidal users (n=259), n (%)	Worried or bereaved users (n=91), n (%)
The counselor seemed warm and caring.	237 (91.5)	87 (95.6)
You felt heard, understood, and respected.	232 (89.5)	87 (95.6)
You were able to express your feelings.	229 (88.4)	86 (94.5)
You talked about what you wanted to talk about.	221 (85.3)	85 (93.4)
You found ways to deal with a particular situation or problem.	200 (77.2)	80 (87.9)
You found that the conversation with the counselor helped you.	217 (83.7)	83 (91.2)
You feel better, calmer, or more relaxed?	214 (82.6)	85 (93.4)
You feel more in control of your suicidal thoughts?	199 (76.8)	—^a^
You feel more able to overcome your difficulties?	188 (72.5)	80 (87.9)

^a^Not available.

## Discussion

### Principal Findings

The objectives of this study were to document the use of the suicide.ca platform; to describe the profile of its users; and to estimate the impact of the universal, selective, and indicated prevention strategies deployed via the platform.

The results for universal prevention strategies indicated that there was a wide range of sources for acquiring traffic to the platform, that actions to promote the platform on social media were directly associated with increased traffic, and that there was little traffic to psychoeducation pages.

The results for selective prevention strategies indicated that AdWords campaigns and fact sheets on suicide risk factors are important sources of traffic acquisition on the platform, that the platform reaches people with varying degrees of suicidal risk, and that the user account was a barrier to the use of mental health self-management tools.

The results for the indicated prevention strategies indicated that triage questions involving personal information result in a significant number of dropouts, that counselors feel that their interventions have a positive impact on the user in half of the contacts, and that intervention service users are generally satisfied with the service they received.

### Universal Prevention Strategies

The sources of traffic acquisition on suicide.ca were very diversified, and only 40% (50,271/125,677) of the users started their visit on the home page. Moreover, the platform’s social media promotion was directly associated with traffic peaks. These results highlight the importance of being proactive in promoting the platform and using a wide variety of promotional strategies (eg, SEO strategy, social media posts, AdWords campaigns, and referral links on other websites). They also highlighted the importance of collaborating with digital experts to adapt traditional digital marketing strategies to suicide prevention.

The number of visits to the psychoeducation pages was far less than the total number of visits to the site. For example, the first page of the psychoeducation section for people with suicidal risk was visited only 5312 times in the first year. This number is far less than the 127,495 visits that were recorded on the site for this period. This result suggests that the vast majority of users do little exploration of the platform after viewing the content that brings them to the site. These results highlight the importance of looking beyond the total number of visits to a digital suicide prevention platform to describe its use and of dissecting its analytics data in detail to fully understand the content that is consulted.

### Selective Prevention Strategies

AdWords campaigns and suicide risk factor fact sheets were important sources of traffic acquisition on the platform. For example, 40% (4704/11,760) of the visits from search engines, excluding AdWords advertisements, began with a suicide risk factor fact sheet. Although it is challenging to determine from the analytical data whether the individuals who accessed the fact sheets or clicked on the advertisements were at risk for suicide, it is evident that they conducted a Google search related to suicide or suicide risk factors and landed on the suicide.ca platform. Furthermore, the AdWords campaigns targeting anxiety and depression achieved click rates of 13.7% and 9.47%, respectively, which are significantly higher than the average Google Ads benchmarks of 3.17% observed in e-marketing [44] and the click rates of 4% and 5% reported in the study by Onie et al [13]. However, despite the high click rates, the conversion rates for crisis chat interventions were relatively low, with 0.38% for the anxiety and depression campaign and 1.93% for the suicide-specific campaign. It is challenging to interpret why these clicks did not result in more crisis interventions. Although the average conversion rate in e-marketing is 3.75% [44], there are currently no established benchmarks for conversion rates in suicide prevention. Although our findings should be interpreted with caution, they offer initial support for suicide.ca’s strategy of using AdWords campaigns and fact sheets as tools to identify internet users presenting suicide risk factors through search engine platforms.

Previous research has predominantly focused on how Google can enhance support for suicide prevention, paying limited attention to how suicide prevention actors can leverage Google to identify individuals at risk (eg, [12,13,45,46]). More research is required to discover optimal methods for using Google search to identify internet users at risk of suicide and to guide them toward support resources.

A major finding of this study is that the platform reaches users with different degrees of suicidal risk. The proportion of users with severe suicidal ideation on the SIDAS-FR was 33.16% (825/2488) for the self-assessment module and 75.56% (1969/2606) for the intervention service. Although the self-assessment module users presented less severe suicidal ideations than the intervention service users, 87.77% (2721/3100) of them obtained a K10 score, indicating the probable presence of a mental health problem. Furthermore, 62.2% (915/1471) of the self-assessment module users obtained a Patient Health Questionnaire-9 score indicating the presence of depression, 61.73% (908/1471) obtained a Penn State Worry Questionnaire score indicating the presence of generalized anxiety disorder, and 53.2% (451/847) obtained a Spin Phobia Inventory score indicating the presence of social phobia disorder.

Our best point of comparison is the 113Online chat service in the Netherlands, which also provides users with access to a self-assessment module and crisis interventions via chat. According to the evaluation of their first 3 years of operation [3], 78.36% (4741/6050) of the users who used the self-assessment module scored positively for depression and anxiety on the Depression and Anxiety Stress Scale. The module also included the Beck Scale for Suicidal Ideation, with results indicating that 7.22% (533/7381) of the users were not suicidal, 1.75% (129/7381) were mildly suicidal, 39.29% (2900/7381) were suicidal, and 51.74% (3819/7381) were severely suicidal. When comparing the results of this study with the observations on the 113Online platform, it appears that the suicide.ca self-assessment module is being used by individuals with lower levels of suicidality. These differences may, in part, be explained by the fact that the 2 platforms used different questionnaires to measure suicidality, depression, and anxiety. In addition, it may be explained in part by the identification strategy of the suicide.ca platform, which aims to reach individuals who have risk factors for suicide but may not be actively suicidal.

With regard to the self-management tools, between 22.99% (1186/5159) and 58% (2992/5159) of the users who downloaded the mobile app did not create a user account. It should be noted that a user can download the app to use the intervention service only and that doing so does not require the creation of an account. Technical problems experienced by users when creating their account could also contribute to this discrepancy [47]. Nevertheless, the discrepancy between the number of downloads and the number of accounts created is so large that it seems to indicate that setting up a user account is a practice that should be avoided as it seems to represent the first barrier to use. This finding highlights an important dilemma for digital suicide prevention interventions. Several studies indicate that users of digital interventions have concerns about the security and privacy of their data [48]. However, although the presence of a user account enhances data security and privacy, it is a barrier to use for some users [49]. These findings highlight the importance of identifying technologies that ensure the security of users’ data while preserving their sense of anonymity.

### Indicated Prevention Strategies

To our knowledge, this study is the first to report on the impacts of triage on the accessibility of a web-based intervention service. Our results indicate that the last question (*What is your first name or alias?*) was associated with a significant number of dropouts; only 46.54% (2711/5825) of the users who were asked the question answered it. Studies on crisis intervention through chat and text indicate that users prefer to use these services over phone helplines because of the sense of anonymity provided by these mediums [3,50,51]. It is possible that even asking for personal information or requesting an alias could discourage users, emphasizing the importance of maintaining their sense of anonymity.

With regard to the questions about suicidality, it is surprising to observe the low dropout rates they have caused. The requirement to complete the 5 questions of the SIDAS-FR during the triage resulted in a dropout rate of 4%. Web-based crisis service providers are sometimes concerned about implementing a triage. This study provides an estimate of the dropout rate that triage can lead to.

With regard to the profile of the intervention service users, most users (2019/3569, 56.57%) were women aged between 18 and 40 years. The most frequently discussed issues were mental health, emotional problems, relationship problems, self-harm, and isolation. These findings are consistent with those typically observed in digital intervention services [14-17].

It is also interesting to note that 7.85% (280/3569) of the users were from gender minority groups and 83.9% (235/280) of them were young people aged ≤25 years. This percentage is similar to that observed in the Canada Suicide Prevention Service (7.5%) and Lifeline Crisis Chat (7.5%) [14,15]. The age of these users likely reflects the high level of adversity experienced at this time for many gender minority youth [52]. These findings also highlight the relevance of digital intervention services for these youth.

This study provides valuable insights into the suicidality of users of chat intervention services, as multiple sources of information were used to evaluate the risk of suicide. These include data derived from a standardized suicide questionnaire (SIDAS-FR), risk assessments conducted by counselors, and a specific question regarding the risk of suicide attempts in the near future. Approximately half of the users (1690/3490, 48.42%) answered *yes* or *uncertain* to the question, *Are you planning to attempt suicide in the coming minutes or hours?* Moreover, 75.56% (1969/2606) of the users who answered *no* or *uncertain* to the question obtained a SIDAS-FR score indicating severe suicidal ideations. Therefore, the intervention service reaches its target population, that is, people at a high risk for suicide. These findings are consistent with those observed in the 113Online crisis chat evaluation [17], where a content analysis of intervention transcripts revealed that 61.1% (320/524) of the users expressed suicidal intentions, 21.2% (111/524) of the users reported both suicidal intentions and a suicide plan, and 3.8% (20/524) of the users were actively attempting suicide at the time of contact.

In terms of the impact of the intervention service, counselors reported that the user felt better after the intervention in 56.09% (2565/4573) of contacts and reported that the user did not feel better in only 9.81% (449/4573) of contacts. The response rate to the postintervention questionnaire was low. Nonetheless, the scores on the various satisfaction indicators were all very high, and 3 (199/259, 76.8%) out of 4 respondents felt that their suicidal ideation had decreased following the intervention. Again, these results are consistent with those typically observed in digital intervention services [15,16,18,53].

### Limitations of the Study

This study had several limitations. First, the results on site traffic, traffic acquisition sources, page views, and app downloads were based on an analysis of the platform’s analytics data. However, analytical data do not allow us to understand the needs and motivations behind users’ browsing behavior on the platform. It is therefore important to complement this type of quantitative data in digital platform evaluations with other observational methods, such as qualitative interviews with users, or by directly observing their browsing and help-seeking behavior on the internet [22].

Similarly, analytical data are often inaccurate. For example, the traffic on the platform was analyzed according to the number of sessions. However, a session is the period during which a user was active on the site. If a user is inactive for ≥30 minutes, Google Analytics will attribute any subsequent activity to a new session. Therefore, the number of sessions is not exactly the same as the number of visits, but it is the closest indicator to it.

With respect to the analysis of app downloads, it was difficult to estimate the number of people who downloaded the app but did not create a user account. Therefore, it was impossible to provide an accurate estimate for this important issue. Moreover, it would be important to understand the needs and motivations that explain why so many people downloaded the app but did not open a user account. We explain this phenomenon by the users’ need for anonymity, but it would be important to validate this hypothesis with empirical evidence.

The results on the needs expressed by the intervention service users and the topics discussed during the contacts were based on the analysis of the intervention reports. The completeness of these reports may vary from one counselor to another, which may explain in part why the numbers on issues discussed were low. Furthermore, data on user-identified needs reflect the perceptions of counselors. Therefore, there is most likely a gap between these perceptions and the actual needs of the users. Therefore, it would be important to conduct qualitative interviews with users to understand their needs and motivations for using the intervention service.

An important limitation of this study was the absence of questions on gender and age in the self-assessment module. Therefore, we were unable to compare users of the self-assessment with users of the intervention service based on these characteristics. This information is crucial to understanding who is and is not being reached by suicide.ca and, consequently, by the Quebec DSPS. This highlights the importance of anticipating how the data collected by the various tools offered on a platform will compare with their users.

### Conclusions

The results of this study indicate that it is possible to use the same platform to conduct universal, selective, and indicated suicide prevention. They also highlighted the importance of being strategic in the development of a digital platform to anticipate how different target populations will be identified, how different intervention modalities will be offered to them, and how the data collected by the platform will allow for the measurement of its impact.

Finally, the evaluation of suicide.ca produced several important results and was the source of a considerable amount of learning. We hope that sharing these learnings will help guide organizations interested in developing a digital suicide prevention platform and contribute to the development of guidance for the development of DSPSs (eg, the WHO [54]).

## Abbreviations

AQPS: Association québécoise de prévention du suicide
CRM: Customer Relationship Management
DSPS: digital suicide prevention strategy
ICT: information and communication technology
K10: Kessler Psychological Distress Scale
SEO: search engine optimization
SIDAS-FR: Suicidal Ideation Attributes Scale—French version
WHO: World Health Organization

## Data Availability

The data sets generated during and analyzed during this study are not publicly available due to confidentiality agreement with the *Association québécoise de prévention du suicide* (AQPS) but are available from the corresponding author on reasonable request.

## References

[1] Rassy J, Bardon C, Dargis L, Côté LP, Corthésy-Blondin L, Mörch CM, Labelle R (2021). Information and communication technology use in suicide prevention: scoping review. J Med Internet Res.

[2] (2014). Preventing suicide: a global imperative. World Health Organization.

[3] Mokkenstorm JK, Huisman A, Kerkhof AJ, Smit JH, Mishara BL, Kerkhof AJ (2013). Results and experiences of 113Online, a comprehensive Dutch online suicide prevention platform. Suicide Prevention and New Technologies.

[4] (2021). Global strategy on digital health 2020-2025. World Health Organization.

[5] It Gets Better home page. It Gets Better.

[6] Gunnell D, Bennewith O, Kapur N, Simkin S, Cooper J, Hawton K (2012). The use of the internet by people who die by suicide in England: a cross sectional study. J Affect Disord.

[7] Mars B, Heron J, Biddle L, Donovan JL, Holley R, Piper M, Potokar J, Wyllie C, Gunnell D (2015). Exposure to, and searching for, information about suicide and self-harm on the internet: prevalence and predictors in a population based cohort of young adults. J Affect Disord.

[8] Mok K, Jorm AF, Pirkis J (2015). Suicide-related internet use: a review. Aust N Z J Psychiatry.

[9] Gunn JF 3rd, Lester D (2013). Using google searches on the internet to monitor suicidal behavior. J Affect Disord.

[10] McCarthy MJ (2010). Internet monitoring of suicide risk in the population. J Affect Disord.

[11] Yang AC, Tsai SJ, Huang NE, Peng CK (2011). Association of internet search trends with suicide death in Taipei City, Taiwan, 2004-2009. J Affect Disord.

[12] Sueki H, Takahashi A, Ito J (2023). Changes in suicide ideation among users of online gatekeeping using search-based advertising. Arch Suicide Res.

[13] Onie S, Berlinquette P, Holland S, Livingstone N, Finemore C, Gale N, Elder E, Laggis G, Heffernan C, Armstrong SO, Theobald A, Josifovski N, Torok M, Shand F, Larsen M (2023). Suicide prevention using Google Ads: randomized controlled trial measuring engagement. JMIR Ment Health.

[14] Côté LP, Mishara BL (2022). Effect of helping suicidal people using text messaging: an evaluation of effects and best practices of the Canadian suicide prevention service's text helpline. Suicide Life Threat Behav.

[15] Gould MS, Chowdhury S, Lake AM, Galfalvy H, Kleinman M, Kuchuk M, McKeon R (2021). National suicide prevention lifeline crisis chat interventions: evaluation of chatters' perceptions of effectiveness. Suicide Life Threat Behav.

[16] Mokkenstorm JK, Eikelenboom M, Huisman A, Wiebenga J, Gilissen R, Kerkhof AJ, Smit JH (2017). Evaluation of the 113Online suicide prevention crisis chat service: outcomes, helper behaviors and comparison to telephone hotlines. Suicide Life Threat Behav.

[17] Pisani AR, Gould MS, Gallo C, Ertefaie A, Kelberman C, Harrington D, Weller D, Green S (2022). Individuals who text crisis text line: key characteristics and opportunities for suicide prevention. Suicide Life Threat Behav.

[18] Williams K, Fildes D, Kobel C, Grootemaat P, Bradford S, Gordon R (2021). Evaluation of outcomes for help seekers accessing a pilot SMS-based crisis intervention service in Australia. Crisis.

[19] Lake AM, Niederkrotenthaler T, Aspden R, Kleinman M, Hoyte-Badu AM, Galfalvy H, Gould MS (2022). Lifeline crisis chat: coding form development and findings on chatters' risk status and counselor behaviors. Suicide Life Threat Behav.

[20] Zabelski S, Kaniuka AR, A Robertson R, Cramer RJ (2023). Crisis lines: current status and recommendations for research and policy. Psychiatr Serv.

[21] Bergin AD, Vallejos EP, Davies EB, Daley D, Ford T, Harold G, Hetrick S, Kidner M, Long Y, Merry S, Morriss R, Sayal K, Sonuga-Barke E, Robinson J, Torous J, Hollis C (2020). Preventive digital mental health interventions for children and young people: a review of the design and reporting of research. NPJ Digit Med.

[22] Rassy J, Mathieu L, Michaud C, Monday T, Raymond S, Bonin JP (2019). The virtual emotional drowning theory: a grounded theory on information and communication technologies (ICT) help-seeking process of adolescents at risk of suicide [Article in French]. Sante Ment Que.

[23] Fleming T, Bavin L, Lucassen M, Stasiak K, Hopkins S, Merry S (2018). Beyond the trial: systematic review of real-world uptake and engagement with digital self-help interventions for depression, low mood, or anxiety. J Med Internet Res.

[24] Shin HD, Durocher K, Sequeira L, Zaheer J, Torous J, Strudwick G (2023). Information and communication technology-based interventions for suicide prevention implemented in clinical settings: a scoping review. BMC Health Serv Res.

[25] Lane J, Côté LP, Gaudreault J, Massicotte L, Manceau LM, Labelle R, Bardon C, Bazinet J, Rassy J, Rembert M (2022). [The development process of the New Quebec Digital Suicide Prevention Strategy: Suicide.ca]. Sante Ment Que.

[26] suicide.ca home page. suicide.ca.

[27] Kessler RC, Barker PR, Colpe LJ, Epstein JF, Gfroerer JC, Hiripi E, Howes MJ, Normand SL, Manderscheid RW, Walters EE, Zaslavsky AM (2003). Screening for serious mental illness in the general population. Arch Gen Psychiatry.

[28] Gauvin G, Bardon C, Côté LP (2022). Psychometric validation of the French version of the Suicidal Ideation Attributes Scale (SIDAS-FR). Death Stud.

[29] (2009). Brief description of the Mental Health Continuum Short Form (MHC-SF). Department of Sociology, Emory University.

[30] Kroenke K, Spitzer RL, Williams JB (2001). The PHQ-9: validity of a brief depression severity measure. J Gen Intern Med.

[31] Hirschfeld RM, Williams JB, Spitzer RL, Calabrese JR, Flynn L, Keck PE Jr, Lewis L, McElroy SL, Post RM, Rapport DJ, Russell JM, Sachs GS, Zajecka J (2000). Development and validation of a screening instrument for bipolar spectrum disorder: the Mood Disorder Questionnaire. Am J Psychiatry.

[32] Weber Rouget B, Gervasoni N, Dubuis V, Gex-Fabry M, Bondolfi G, Aubry JM (2005). Screening for bipolar disorders using a French version of the Mood Disorder Questionnaire (MDQ). J Affect Disord.

[33] Connor KM, Davidson JR, Churchill LE, Sherwood A, Foa E, Weisler RH (2000). Psychometric properties of the Social Phobia Inventory (SPIN). New self-rating scale. Br J Psychiatry.

[34] Gosselin P, Dugas MJ, Ladouceur R, Freeston MH (2001). Evaluation of worry: validation of a French translation of the Penn State Worry Questionnaire [Article in French]. Encephale.

[35] Meyer TJ, Miller ML, Metzger RL, Borkovec TD (1990). Development and validation of the Penn State Worry Questionnaire. Behav Res Ther.

[36] Tremblay J, Blanchette-Martin N (2016). Manuel d'utilisation du DÉBA-Alcool/Drogues/Jeu-8-Version adaptée pour la formation de la première ligne en dépendance. Integrated University Health and Social Services Center of the Capitale-Nationale.

[37] Dufour M, Tremblay J, Blanchette-Martin N, Ferland F, Goyette M, Turcotte S, Khazaal Y, Brunelle N, Gagnon SR, Tétrault-Beaudoin CS, Genois R, Légaré AA (2019). Dépistage/Évaluation du Besoin d'Aide - Internet (DÉBA-Internet). Université de Sherbrooke.

[38] Weathers FW, Litz BT, Keane TM, Palmieri PA, Marx BP, Schnurr PP (2013). The PTSD Checklist for DSM-5 (PCL-5). National Center for Posttraumatic Stress Disorder.

[39] Caron J (1996). The scale of social provisions: their validation in Quebec [Article in French]. Sante Ment Que.

[40] Cutrona C, Russell DW, Jones WH, Perlman D (1983). The provisions of social relationships and adaptation to stress. Advances in Personal Relationships.

[41] Labelle R, Lachance L, Morval M (1996). Validation d'une version canadienne-française du reasons for living inventory. Sci comport.

[42] Linehan MM, Goodstein JL, Nielsen SL, Chiles JA (1983). Reasons for staying alive when you are thinking of killing yourself: The Reasons for Living Inventory. J Consult Clin Psychol.

[43] Clifton B (2010). Advanced Web Metrics with Google Analytics.

[44] Irvine M Google Ads benchmarks for your industry updated!. WorldStream.

[45] Liu NH, Contreras O, Muñoz RF, Leykin Y (2014). Assessing suicide attempts and depression among Chinese speakers over the internet. Crisis.

[46] Sueki H (2015). Suicide prevention using the internet: mini-review and a case study in online gatekeeping activity. Suicidal Ideation: Predictors, Prevalence and Prevention.

[47] Balaskas A, Schueller SM, Cox AL, Doherty G (2022). Understanding users' perspectives on mobile apps for anxiety management. Front Digit Health.

[48] Chan AH, Honey ML (2022). User perceptions of mobile digital apps for mental health: acceptability and usability - an integrative review. J Psychiatr Ment Health Nurs.

[49] Alqahtani F, Winn A, Orji R (2021). Co-designing a mobile app to improve mental health and well-being: focus group study. JMIR Form Res.

[50] Hanley T (2011). Understanding the online therapeutic alliance through the eyes of adolescent service users. Couns Psychother Res.

[51] Navarro P, Bambling M, Sheffield J, Edirippulige S (2019). Exploring young people's perceptions of the effectiveness of text-based online counseling: mixed methods pilot study. JMIR Ment Health.

[52] Clements-Nolle K, Marx R, Katz M (2006). Attempted suicide among transgender persons. J Homosex.

[53] Sindahl TN, Côte LP, Dargis L, Mishara BL, Bechmann Jensen T (2019). Texting for help: processes and impact of text counseling with children and youth with suicide ideation. Suicide Life Threat Behav.

[54] (2018). National suicide prevention strategies: progress, examples and indicators. World Health Organization.

